# Stroke Steadiness as a Determinant Factor of Performance in 100 m Freestyle in Young Swimmers

**DOI:** 10.3390/sports12040107

**Published:** 2024-04-12

**Authors:** Daniel López-Plaza, Carmen Daniela Quero-Calero, Fernando Alacid, Oriol Abellán-Aynés

**Affiliations:** 1 Department of Education, Health Research Center, University of Almeria, 04120 Almeria, Spain; dlplazapal@ual.es (D.L.-P.); falacid@ual.es (F.A.); 2 International Chair of Sport Medicine, UCAM Catholic University of Murcia, 30007 Murcia, Spain; 3 Faculty of Sport, UCAM Catholic University of Murcia, 30007 Murcia, Spain; 4 Department of Health Sciences, Public University of Navarre (UPNA), 31008 Pamplona, Spain; abellanoriol@gmail.com

**Keywords:** biomechanics, kinematics, performance, variability

## Abstract

The classical kinematic variables in swimming are based on the calculation of mean values. Stroke steadiness determines the relationship between the duration of all consecutive strokes throughout a test. The aims of the current investigation were to examine differences in stroke-to-stroke steadiness according to swimmers’ performance level on both body sides (breathing and non-breathing) and to analyse the interrelationship with kinematics during a 100 m front-crawl test. Thirty-two young, experienced swimmers voluntarily participated in the present study and were divided into two groups, national level (*n* = 15) and local level (*n* = 17), according to their competitive status within the national age-rankings. All participants performed a 100 m maximal test in a 50 m pool where they were laterally recorded. Kinematic variables such as mean velocity, stroke rate, stroke length, and stroke index, as well as long-term steadiness and short-term steadiness, were calculated. The two 50 m sections were analysed independently. Significant differences were observed between the two groups in the classical kinematic variables and in stroke steadiness (*p* < 0.05). In addition, stroke steadiness showed moderately high correlations with velocity (r = [−0.61–(−0.749)]) and stroke index (r = [−0.356–(−0.582)]). Maintaining a more stable inter-stroke period appears to be a determinant of performance in young, high-level national swimmers.

## 1. Introduction

The ability of a competitive swimmer to attain and maintain optimal swimming velocity is determined by the interaction of energetics and kinematics. The maximum velocity can be expressed as the balance between maximal total energy expenditure and energy cost [1,2]. Since previous research has identified drag forces as a major determinant to energy cost [1,3], the technical capacity of swimmers is especially important for an efficient performance in all four strokes [4]. For a given velocity, alternated strokes (freestyle and backstroke) are more economical than the simultaneous ones (butterfly and breaststroke) [5]. In addition, higher-level sprint swimmers exhibit greater propulsive efficiency [6,7,8], suggesting that optimal swimming technique contributes to better propelling through the water by increasing mechanical work effectiveness.

The classic kinematic analysis in swimming has traditionally included not only the stroke rate (SR) and stroke length (SL) as a determinants of swimming velocity, but also the stroke index (SI) as a measure of biomechanical efficiency [9,10,11]. Prior investigations have identified SL as the key determinant to performance in swimming since more successful competitors at all ages are able to stabilise it for longer through the race, resulting in higher SI [12,13,14,15,16,17]. Typically, in short-distance events, SR tends to decrease while swimmers struggle to maintain a large SL in an attempt to reduce velocity decreases [16,18,19,20].

In swimming and other cyclic sports, classical kinematic variables have always been measured by mean values, representing with one value all the strokes during a specific test regardless of the relationship between each of them. However, it has recently been proposed that the analysis of variations between consecutive cycles can directly influence performance in other cyclic water sports such as canoeing [21]. For this reason, it seems interesting to test whether more variable or steadier periods between strokes will have a direct effect on the swimmer’s velocity and, therefore, on performance. In fact, some recent studies have aimed to investigate whether instantaneous variations in velocity can have a direct effect on total performance in swimmers [22].

In the last decades, the improvement in the technology of video analysis and accelerometry has favoured the appearance of additional kinematic parameters that have been successfully applied to better understand the propelling efficiency in swimming [13,17,23,24]. The intra-cycle velocity variation represents the variation of the horizontal velocity of the centre of mass and, theoretically, is related to a lower energy cost as a result of propulsive continuity [6,25]. In young swimmers, intracyclic velocity variations were identified as a predictor of swimming performance [26]. However, the relationship between IVV and velocity in senior swimmers is still controversial among researchers [1,6,22]. In alternated stroke styles such as freestyle, the co-ordination between the propulsive phases of the stroke can be described by the index of co-ordination (IdC). According to swimming level and distance, a higher and more stable IdC (superposition co-ordination) were observed in elite 100 m competitors and were associated with more consistent and continuous propulsion [1,13,16,17]. What has been described so far in other cyclic sports is that the steadier the time between cycles, the lower the speed losses, resulting in a higher performance [21]. In addition, the breathing pattern is a factor influencing kinematics in front-crawl swimming, observing differences in stroke co-ordination indices between the breathing and non-breathing sides [27,28]. However, to the best of our knowledge, the independent kinematic analysis according to body sides while following the preferred breathing pattern is not yet studied in swimming.

Apparently, the definition and application of new parameters such as stroke steadiness is required to better understand the relationship of the temporal aspects of the swimming cycle with velocity variations and propulsion efficiency. Therefore, the aims of the current investigation were: (1) to identify the differences in stroke-to-stroke time steadiness according to swimmers’ performance level on both body sides (breathing and non-breathing); and (2) to determine the interrelationship with kinematics during a 100 m front-crawl test. It was hypothesised that higher-performance swimmers would exhibit a higher stroke steadiness than lower-performance ones, along with higher swimming speeds.

## 2. Materials and Methods

### 2.1. Participants

Thirty-two young and experienced swimmers (17 boys and 15 girls), with a training volume of at least 15 h/week, voluntarily participated in the present study. Although not all swimmers were front-crawl specialists, at least half of their training volume was performed with front crawl as primary or secondary stroke technique. All participants were assigned to one of the two groups based on their competitive level within their national age-ranking. The national-level group (G1) was composed of 15 swimmers (9 boys and 6 girls) in the top 10 of the national ranking (age: 15.55 ± 1.49 years; training experience: 8.31 ± 2.97 years; FINA points in 100 m free: 651.25 ± 46.56) that were also selected by the Regional Federation to participate in a Development Program as the best in their age groups. A total of 17 local swimmers (8 boys and 9 girls) that belonged to 3 different swimming clubs of the surrounding area (age: 15.91 ± 1.93 years; training experience: 6.35 ± 2.32 years; FINA points in 100 m free: 503.50 ± 64.95) were allocated to the local-level group (G2)

After an informative session explaining the procedures of the study, a written informed consent form was required to be completed by all participants and their parents/guardians. Any participant presenting signs of disease or under pharmacological treatment was excluded from testing. The study was approved by the university’s Ethics Committee (protocol code: 241011). In addition, the procedures described below followed the Declaration of Helsinki.

### 2.2. Study Design

In a 50 m pool, all swimmers performed a 100 m front crawl at maximum effort starting from the water and after a visual–acoustic signal. Participants were instructed to breathe only by their preferred side. An experienced operator recorded each individual performance by a high-speed video camera (GoPro Hero 5, GoPro Inc., San Mateo, CA, USA) at 120 frames per second. To avoid parallax errors, the camera was fixed to a mobile trolley that followed the head of the swimmer along the course. Only the 20 central meters of the swimming course were used for the analysis of basic kinematic variables to prevent the influence of the start and the turn. Four external markers were allocated on both sides of the pool for that purpose, two at 15 m and two at 35 m (two markers on each side), allowing the caption of the head in line with the markers during the tests.

### 2.3. Data Analysis

Frame-by-frame recordings of each participant were examined using Virtualdub software 1.10.4 (Avery Lee). Basic kinematic parameters (swimming velocity, V; stroke rate, SR; stroke length, SL; and stroke index, SI) were obtained in the central 20 m of each 50 m section using the equations described by Craig et al. [10] and Costill et al. [9]: SL = V · SR and SI = V · SL. The start and end of a stroke were established by the frame when the hand of the same side contacted the water in subsequent strokes. In this way, strokes for the left and right hand were analysed independently. Two consecutive strokes of the same arm were considered a cycle. To calculate V, the frames taken to cover the 20 m distance (m · s^−1^) were examined, whereas SR was obtained in cycles · min^−1^ based on the frame difference between 3 consecutive strokes of the same side. SL and SI were extrapolated from the equations described above and represented in m · cycle and in cycles · m^2^ · s^−1^, respectively. In addition, basic kinematic parameters according to each body side were also calculated to examine the differences in the breathing and non-breathing side.

Furthermore, every single stroke during the test was analysed (breathing and non-breathing side independently), obtaining the period between all the strokes and the one immediately following each stroke. By analysing all the periods (*S*) during the test, a time series of the type {S__1_…S__n_…S__N_} was obtained where *N* is the total number of strokes for each participant. To analyse the steadiness of the strokes, long- and short-term variations were studied. The long-term steadiness (*LTS*) was calculated based on the standard deviation of the periods between consecutive strokes as:

(1) $$LTS=\sqrt{\frac{\sum_{i}^{N}{\left(S_{i}-S\right)}^{2}}{N}}\;$$

while the short-term steadiness (*STS*) analysis was based on the root-mean-square of successive differences between strokes in order to obtain a value of instantaneous variation between strokes following Equation (2):

(2) $$STS=\sqrt{\frac{\sum_{i=1}^{N-1}{\left(S_{i}-S_{i+1}\right)}^{2}}{N-1}}\;$$

### 2.4. Statistical Analysis

Mean and standard deviation (SD) of all variables were analysed using the statistical package SPSS version 24 (IBM, New York, NY, USA) for windows. To examine the hypothesis of normal distribution and homogeneity of variance, Shapiro–Wilk and Levene’s test were conducted, respectively. A *t*-test for independent samples was used to examine the differences between groups when no violations of the assumptions of normality and homogeneity were detected. If normality supposition of data was rejected, Mann–Whitney U-test was performed. To analyse the differences between the first and last 50 m section, a *t*-test for paired samples was used when variables followed a normal distribution. If normality of data was rejected, a Wilcoxon test was performed. For all tests, the level of significance was set at 0.05. In addition, the effect size of the comparison between G1 and G2 was calculated through Cohen’s D, as well as the upper and lower 95% confidence level for effect size. Effect sizes were interpreted as very low when lower than 0.2; low when ranged between 0.2 and 0.5; moderate when ranged between 0.5 and 0.8; and high when it was higher than 0.8. The effect size was considered significantly high when the value zero was not in the range between upper and lower 95% confidence interval. Pearson’s correlation coefficient (r) or Spearman’s correlation coefficient (rs) when normality supposition was violated were conducted to determine the interrelationship between stroke steadiness and basic kinematic variables. Correlation values were interpreted as none (r < 0.2), low (0.2 < r < 0.4), moderate (0.4 < r < 0.6), high (0.6 < r < 0.8), and very high (r > 0.8), consequently, for negative correlations.

## 3. Results

Table 1 summarises the kinematics comparison between G1 and G2 in both 50 m sections of the 100 m independently for the breathing side. Significant greater kinematic values were identified in the national level group, especially in the second 50 m (*p* < 0.05), whereas stroke steadiness variables also revealed significant inter-group differences in *LTS* and *STS* values.

The results for the non-breathing side comparing performance groups are presented in Table 2; the results of the first and the second half of the test are presented separately. Similarly, significant differences between groups were observed in velocity in both sections, as well as in most classical kinematic and steadiness variables (*p* < 0.05).

Table 3 shows the r values of the correlation between the steadiness and kinematic variables for the breathing side. Moderately high correlation values from −0.63 to −0.74 were identified between steadiness variables (*STS* and *LTS*) and V and SI in both 50 m sections, while no consistent values of correlation with SF and SL were observed.

The r values of the correlation between the steadiness and the classical kinematic variables for the non-breathing side are presented in Table 4. *LTS* and *STS* were negatively associated with V and SI, especially in the second and the first 50 m sections, respectively. Conversely, less consistent value interrelationships were detected with SF and SR.

## 4. Discussion

The main aims of this paper were to identify the differences in stroke-to-stroke time steadiness according to swimmers’ performance level and to determine its interrelationship with kinematics during a 100 m front-crawl test. The main finding of the current investigation was the significantly greater stroke steadiness exhibited by the more successful swimmers in both the breathing and the non-breathing side of a 100 m front crawl. Although, in both 50 m sections, significant differences between groups were detected in stroke steadiness and kinematic variables, larger differences were observed in the second 50 m. National-level swimmers not only exhibited more stable swimming strokes but also performed faster and more efficient propulsions according to the SL, SI, and V values. In addition, stroke steadiness was associated with V, suggesting the relevance of this parameter as a determinant of swimming performance.

Stroke steadiness is a relatively new kinematic parameter described for the first time by Abellán-Aynés et al. [21] in sprint canoeing as a measure of variability of SR. Apparently, the reduction of time variations between consecutive strokes in canoeing were strongly associated with boat speed as a result of strokes of similar duration and continuous propulsion during a race [21]. In a recent study, Ganzevles et al. [29] reported that faster swimmers performed smoother strokes by reducing jerk actions during the arm cycle in the 50 m all-out test. Similar results were observed in the current investigation, as stroke steadiness was significantly related to swimming velocity.

In contrast with the SR results obtained here, stroke steadiness in national-level swimmers showed a significantly higher stability between consecutive strokes. This is especially representative since SR only provides the number of strokes in a certain time [10], whereas stroke steadiness represents the stroke-to-stroke variations and duration in time [21]. The analysis between body sides revealed that local-level swimmers struggled to maintain stroke steadiness on both sides, but particularly on the breathing side. Similarly, Seifert et al. [28] reported a significantly greater catch-up co-ordination in the strokes of the breathing side while swimming using a preferred-two-strokes breathing, suggesting a lower propulsive continuity on that side. Taking into consideration that the co-ordination between stroke phases is related to a constant velocity, [23] maintaining stroke co-ordination while breathing in freestyle seems paramount for an optimal swimming performance.

Although pacing strategies in sprint swimmers have been widely studied, SR is largely dependent on individual characteristics, and swimmers with different pacing preferences could be equally successful in a given race [10,14,30,31]. Nevertheless, stroke steadiness seemed to be related to the consistency of strokes and, consequently, to the application of propulsive forces. Propulsive continuity has traditionally been related to low intra-cycle velocity variations, resulting in a lower energy cost. [6] However, contradictory results about the relationship between intra-cycle velocity variation and swimming velocity have been reported in previous investigations [1,6]. Thus, these evidences might suggest that other phenomena such as inter-stroke rate variations defined by stroke steadiness might be a better determinant of swimming performance.

A noteworthy aspect is that, although no changes in SR were detected between performance groups, maintaining a constant SR, which means increasing stroke steadiness, does lead to a 100 m performance improvement. *LTS* refers to the variation between all the strokes performed during the course while *STS* is associated with the variation between one stroke and the immediately subsequent one. Observing the relationship of both with V, stroke steadiness seems to be a determinant for speed maintenance throughout a race. Furthermore, *LTS* showed that the sequence of strokes throughout the trial translates into higher performance when the sequence is less variable. In turn, smaller variations between consecutive strokes are also identified to be a fundamental aspect in maintaining velocity during the test. Similarly, both variables revealed a moderate correlation with SI, which might indicate that *LTS* and *STS* are closely related to higher efficiency during 100 m tests.

Although both *STS* and *LTS* exhibited similar relationships with V and SI, relatively higher correlation values were determined in *LTS*, especially with SI. In addition, greater differences between G1 and G2 were observed in *LTS* than in *STS* for both breathing sides and in both sections of the course. This aspect was also observed in short canoe distances [21], where the authors highlighted the slightly higher relationship of *LTS* with the boat speed. Therefore, not only does a higher steadiness between consecutive strokes seem to increase V in these cyclic sports, but maintaining a similar instantaneous SR throughout the test was also identified as a slightly more critical factor in achieving higher V values.

Since great SL and SI have been commonly identified among high-level competitors, they can be considered as determinants for optimal swimming performance in long [14,32] and short distances [8,17,26,33]. The greater SL and SI values observed in both breathing and non-breathing sides in national compared to local-level competitors were in agreement with the previous research analysing young swimmers’ performance in short distances [26,34]. In addition, SL seems especially important for V and SI maintenance in the last section of a race where most successful swimmers are characterised by minimising the typical decreases in distance per stroke. Stroke steadiness was significantly associated with SI, observing greater values in the second half of the 100 m. Perhaps the stroke efficiency associated with these parameters might be related to the ability to maintain more stable propulsions in consecutive strokes. 

As far as the authors know, this is the first study investigating inter-stroke steadiness in swimming. The results of the current investigation might provide us with normative data about the influence of stroke steadiness in swimming performance. For coaches and swimming analysts, knowing these specific biomechanical aspects might help us to better understand the influence of time variations between strokes in competitive swimming. In terms of application, coaches might consider including some sort of technical drills and workouts within their training programs for swimmers to internalise more consistent strokes, knowing that instantaneous changes in pace could lead to reductions in average velocity over short distances. Furthermore, understanding this factor as a determinant of performance will result in devices that can directly quantify stroke steadiness to facilitate analysis and, therefore, make it more applicable to training by swimming coaches. Once this has been achieved, this variable can be used to quantify improvements derived from strength training or athlete maturation and its influence on performance.

The fact that boys and girls were examined without distinguishing between the sexes might be considered a limitation in the present investigation. However, there is evidence of no differences in kinematics’ tendency in sprint events between sexes [17]. In addition, all participants were pubertal swimmers and, taking into consideration the early maturation by girls during adolescence [35], girls might overcome the physical capacity differences observed later during adulthood. Another limitation is related to the point of reference of the start and end of the stroke. It would have probably been more accurate to consider the start of the stroke as the moment when the propulsion phase begins by an underwater recording, an aspect that should be taken into account for future research on the analysis of stroke steadiness in swimming. Furthermore, to improve the accuracy of the analysis, accelerometry should be used for this type of study to quantify the acceleration points in the strokes; however, there are no validated devices available to date to quantify stroke steadiness using these methods.

## 5. Conclusions

The kinematic variables studied to date have been of great use in understanding and improving swimming performance. However, the application of new types of kinematic variables such as stroke steadiness might help to better understand the stroke determinants of swimming performance. The current research revealed that greater steadiness within consecutive strokes in 100 m front-crawl style events was a determining factor for attaining high velocity in elite young swimmers. Furthermore, more successful swimmers tended to maintain stroke steadiness in the second 50 m section better on both body sides, but especially in the non-breathing side. Therefore, the reduction of variability between strokes appears to be fundamental for the improvement of swimming performance and, possibly, in other sports that involve a cyclical approach.

## Figures and Tables

**Table 1:** Comparison between national-level and local-level groups in kinematic and steadiness variables on breathing side.

Outcome	G1 (*n* = 15)		G2 (*n* = 17)		*p*	ES [LCI, UCI]
	Mean	SD	Mean	SD		
**0–50 m**						
V (m·s^−1^)	1.63	0.14	1.43	0.08	**0.008**	**1.78 [0.92, 2.55]**
SF (cycles·min^−1^)	49.45	3.42	48.09	3.84	0.480	0.37 [−0.33, 1.06]
SL (m·cycle)	1.99	0.22	1.80	0.14	0.08	**1.04 [0.28, 1.75]**
SI (cycles·m^2^·s^−1^)	3.27	0.63	2.60	0.30	**0.023**	**1.38 [0.58, 2.12]**
*LTS* (ms)	49.98	12.76	82.25	43.05	**0.039**	**−0.98 [−1.69, −0.23]**
*STS* (ms)	47.63	29.49	78.43	55.07	0.095	−0.68 [−1.38, 0.04]
**50–100 m**						
V (m·s^−1^)	1.56	0.11	1.32	0.08	**<0.001**	**2.52 [1.53, 3.37]**
SF (cycles·min^−1^)	47.39	2.80	44.91	3.20	0.133	**0.82 [0.07, 1.52]**
SL (m·cycle)	1.98	0.17	1.77	0.14	**0.024**	**1.35 [0.55, 2.08]**
SI (cycles·m^2^·s^−1^)	3.13	0.47	2.35	0.30	**0.003**	**2 [1.11, 2.79]**
*LTS* (ms)	35.43 *	14.79	56.00 *	17.86	**0.03**	**−1.24 [−1.97, −0.45]**
*STS* (ms)	41.11	11.93	61.44	23.63	**0.037**	**−1.06 [−1.77, −0.29]**

* *p* < 0.05 compared to 0–50 m. Abbreviations: G1 = national-level group; G2 = local-level group; SD = standard deviation; V = velocity; SF = stroke frequency; SL = stroke length; SI = stroke index; *LTS* = long-term steadiness; *STS* = short-term steadiness; ES: effect size; LCI: lower confidence interval for effect size; UCI: upper confidence interval for effect size. Bold: significant values.

**Table 2:** Comparison between national-level and local-level group in kinematic and steadiness variables on non-breathing side.

Outcome	G1 (*n* = 15)		G2 (*n* = 17)		*p*	ES [LCI, UCI]
	Mean	SD	Mean	SD		
**0–50 m**						
V (m·s^−1^)	1.63	0.14	1.43	0.08	**0.008**	**1.78 [0.92, 2.55]**
SF (cycles·min^−1^)	49.54	3.39	54.95	19.21	0.445	−0.38 [−1.07, 0.32]
SL (m·cycle)	1.98	0.22	1.67	0.34	0.053	**1.06 [0.3, 1.78]**
SI (cycles·m^2^·s^−1^)	3.26	0.63	2.39	0.48	**0.011**	**1.56 [0.73, 2.31]**
*LTS* (ms)	56.19	12.41	83.12	30.20	**0.024**	**−1.13 [−1.85, −0.36]**
*STS* (ms)	55.97	32.51	98.38	67.66	**0.041**	**−0.78 [−1.48, −0.04]**
**50–100 m**						
V (m·s^−1^)	1.56	0.11	1.32	0.08	**<0.001**	**2.52 [1.53, 3.37]**
SF (cycles·min^−1^)	49.40	3.39	48.05	3.82	0.456	0.37 [−0.33, 1.06]
SL (m·cycle)	1.91	0.18	1.66	0.14	**0.014**	**1.56 [0.73, 2.31]**
SI (cycles·m^2^·s^−1^)	3.04	0.48	2.20	0.28	**0.002**	**2.17 [1.25, 2.98]**
*LTS* (ms)	53.63	10.56	83.09	36.77	**0.032**	**−1.05 [−1.77, −0.29]**
*STS* (ms)	50.48	32.06	85.53	49.46	**0.039**	**−0.82 [−1.53, −0.08]**

Abbreviations: G1 = national-level group; G2 = local-level group; SD = standard deviation; V = velocity; SF = stroke frequency; SL = stroke length; SI = stroke index; *LTS* = long-term steadiness; *STS* = short-term steadiness; ES: effect size; LCI: lower confidence interval for effect size; UCI: upper confidence interval for effect size. Bold: significant values.

**Table 3:** Correlation (r) of steadiness variables with kinematic variables in the first and last 50 m of the 100 m test in breathing side (*n* = 32).

	V	SF	SL	SI
**0–50 m**				
*LTS*	**−0.682**	−0.077	−0.222	**−0.582**
*STS*	**−0.629**	0.004	−0.132	−0.442
**50–100 m**				
*LTS*	**−0.741**	**−0.570**	0.129	**−0.534**
*STS*	**−0.614**	−0.184	−0.06	−0.356

Abbreviations: *LTS* = long-term steadiness; *STS* = short-term steadiness; V = velocity; SF = stroke frequency; SL = stroke length; SI = stroke index. Bold: significant values.

**Table 4:** Correlation (r) of steadiness variables with kinematic variables in the first and last 50 m of the 100 m test in non-breathing side (*n* = 32).

	V	SF	SL	SI
**0–50 m**				
*LTS*	**−0.682**	0.401	−0.472	**−0.559**
*STS*	**−0.610**	**0.656**	**−0.557**	**−0.534**
**50–100 m**				
*LTS*	**−0.749**	−0.064	−0.298	**−0.444**
*STS*	**−0.735**	−0.063	−0.192	**−0.419**

Abbreviations: *LTS* = long-term steadiness; *STS* = short-term steadiness; V = velocity; SF = stroke frequency; SL = stroke length; SI = stroke index. Bold: significant values.

## Data Availability

The data that support the findings of this study are available from the corresponding author upon reasonable request. The data are not publicly available due to ethics and data protection of participants.
